# Peripheral Intravenous Catheterisation in Obstetric Patients in the Hand or Forearm Vein: A Randomised Trial

**DOI:** 10.1038/srep23223

**Published:** 2016-03-18

**Authors:** Peng Chiong Tan, Anjana Mackeen, Su Yen Khong, Siti Zawiah Omar, M. A. Noor Azmi

**Affiliations:** 1 Department of Obstetrics and Gynaecology, Faculty of Medicine, University of Malaya, Lembah Pantai, 50603 Kuala Lumpur, Malaysia

## Abstract

A peripheral intravenous catheter is often inserted as part of care during labour. The catheter is inserted into the back of the hand or lower forearm vein in usual practice. There is no trial data to guide the care provider on which is the better insertion site in any clinical setting. 307 women admitted to the labour ward who required insertion of intravenous catheter were randomised to back of hand or lower forearm vein catheter insertion. Catheter insertion is by junior to mid-grade providers. We evaluated insertion success at the first attempt, pain during insertion and catheter replacement due to malfunction as main outcomes. After catheter removal, we recorded patient satisfaction with site, future site preference and insertion site swelling, bruising, tenderness, vein thrombosis and pain. Insertion of a catheter into back of hand vein is more likely to be successful at the first attempt. Insertion pain score, catheter replacement rate, patient satisfaction, patient fidelity to site in a future insertion and insertion site complications rate are not different between trial arms. In conclusion, both insertion sites are suitable; the back of the hand vein maybe easier to cannulate and seems to be preferred by our frontline providers.

Intravenous catheterisation in women admitted to the delivery suite is common due to linked practical considerations: for blood sampling, in anticipation of frequently needed intravenous hydration, antibiotics or oxytocin during labor and as a precaution in the case of hemorrhage.

Clinical trials on peripheral intravenous catheterisation have focused on catheter longevity by evaluating timing of replacement^1^ and intermittent flushing or infusion with or without heparin to maintain patency^2^,^3^,^4^, inline filters to reduce phlebitis^5^, timing of giving set changes^6^, various pain relief methods during insertion^7^,^8^,^9^,^10^,^11^,^12^,^13^,^14^, catheter dressings^15^ and even local warming to aid insertion^16^. Despite the ubiquitous presence of the peripheral intravenous catheters in women on many delivery suites, the very few published trials in obstetric patients have focused on catheter patency^17^,^18^. In obstetric patients anatomic considerations can be different as distal veins are engorged counterbalanced by peripheral oedema of late pregnancy and catheters are often needed for a short period only. In these patients, insertion success, insertion pain and short-term catheter functionality are more relevant. Senior providers on our delivery suite encouraged catheter insertion at the forearm rather than dorsum of the hand vein based on the belief lower forearm insertion is just as likely to be successful^19^ and less painful, infusant flow is better as the vein is larger or the catheter tip is not blocked by wrist movement, the catheter is easier to secure and sits more comfortably and the anaesthetist is less likely to insert an additional catheter if anaesthesia is needed for an unplanned procedure. We performed a pilot study, then a powered trial to test our hypothesis on the impact of catheter site (dorsum of the hand vs. lower forearm) in delivery suite patients on insertion success, insertion pain and catheter replacement due to malfunction.

## Methods

Ethical approval for the trial was obtained from the University of Malaya Medical Center Medical Ethics Committee (approval number 975.12 on 12 March 2013). The study was conducted in the Obstetric Unit of University Malaya Medical Center, Kuala Lumpur, Malaysia and in accordance with Declaration of Helsinki. The trial was registered in a public trial registry (registration number ISRCTN62901900; 7 May 2014) before the start of enrolment.

To help develop the trial protocol, we first conducted a pilot observation of 100 consecutive routine peripheral intravenous catheterisations in our delivery suite to gauge the insertion success rate at common insertion sites. Delivery suite providers were asked to document the site of their first catheter insertion attempt and whether it succeeded. There were 81 first attempts on dorsum of the hand vein and 19 on forearm (typically just proximal to the wrist) vein and insertion success rates were 72/81 (89%) and 14/19 (74%) respectively (Relative risk RR 1.2 95% CI 0.9–1.6 P** = **0.188). These initial findings demonstrated a marked tendency amongst junior providers to use dorsum of the hand vein in their routine practice, plausibly motivated by the perceived ease of catheter insertion there.

Our sample size was calculated based on a superiority hypothesis using our pilot data insertion success rates of 89% vs. 74% for dorsum of the hand vs. forearm veins respectively: taking alpha of 0.05, power of 90% and 1 to 1 enrolment ratio, 139 participants were needed in each arm^20^. We added 10% to that base number to cater for potential drop-outs resulting in a final number of 152 participants needed in each arm (total 304). We assumed that a 1 point difference in insertion pain to be clinically relevant, with pain measured using a 10 point visual numerical rating scale (VNRS scored 1–10, high score more pain) and estimated the standard deviation of the pain VNRS to be 2.5: applying these assumptions and taking alpha 0.05, power at 90%, 1 to 1 enrolment ratio, using the Student t-test and adding a 10% margin for drop outs, 145 participants are needed in each arm (total 290)^20^.

The randomisation sequence is generated through random.org in random blocks of 8 or 12 by an author PCT who was not involved in enrolment. A total of 320 envelopes sequentially numbered were prepared (comprising 16 blocks of 8 and 12 each). These numbered envelopes were arranged in sequence and placed in a trial box kept on the main station of the delivery suite. The allocated instruction for dorsum of the hand or forearm vein insertion was placed in a smaller envelope concealed within the larger numbered sealed envelope which also contained the case report form, numerical rating scales and questionnaires. Randomisation is effected by the opening of the next numbered envelope remaining in the trial box. We believe there was no practical way to mask the trial interventions or in outcomes assessment.

Midwifery and medical providers who worked on the delivery suite and postnatal ward during trial enrolment were briefed and instructed at least weekly about the trial protocol. In our delivery suite, peripheral intravenous catheterisation is routinely performed by house officers or medical officers. House officers are new medical graduates undergoing a compulsory 2-year preregistration training program which includes an obligatory 4-month rotation in obstetrics and gynaecology. Our medical officers are fully registered doctors in our 4-year specialty training program.The provider (house officer or medical officer) who inserts the intravenous catheter was required to have a minimum of six months experience with peripheral intravenous catheterisation and permitted independent practice of the procedure. Women admitted to the delivery suite who according to our usual practice need to have a peripheral intravenous catheter inserted, were aged 18 years or older, at term gestation (>36 weeks) with a singleton viable pregnancy and who had suitable veins at both the back of the hand and the lower forearm for catheter insertion were invited to participate. We exclude women who were judged unstable possibly requiring multiple venous accesses or considered to require immediate Caesarean delivery (our anaesthesia colleagues preferred the forearm site). The eligible woman was provided with the patient information sheet and counseled by the provider who obtained written informed consent from all who agreed to participate. Patient characteristics, indications for intravenous catheterisation and participants’ catheter site preference (before insertion) were collected.

In our delivery suite, the winged 18G (45 mm length) intravenous over-the-needle catheter with injection and main ports (Vasofix^®^ Branule^®^ B Braun Melsungen AG, Germany) was the standard catheter in use, usually inserted into the dorsum of the hand or lower forearm vein. This was the study catheter. Blood sampling through the catheter main port is only permitted at the time of insertion. Local anesthesia was not routine for the insertion of the 18G catheter.

Within the trial, the 18G catheter was inserted according to the provider’s usual technique after skin cleaning without local anesthesia [which can impact insertion success^21^ and its administration can also cause pain^7^]. The provider was instructed to insert in the non-dominant arm if a suitable vein was available and to avoid veins at the antecubital fossa or upper forearm where the catheter tip will extend to the elbow crease after insertion. Blood was withdrawn from the catheter main port if needed and a spigot applied, catheter secured with the standard transparent plaster dressing and the catheter flushed with 5 ml of normal saline. The provider then self-graded the insertion difficulty. After the first insertion attempt, whether successful or not, the participant was asked to rate the insertion associated pain using a VNRS (scored 1 to 10; high score more pain). We recorded any subsequent catheter replacement due to indwelling catheter malfunction (and its specific causes). The catheter insertion success rate, insertion pain score and catheter replacement rates were the a priori primary outcomes of this trial.

If the insertion failed, there was inability to aspirate blood from the main catheter port (if needed) despite manipulation to avoid venous valves or concern about correct placement during the saline flush, the catheter was removed and the attempt was classified as a failure. The provider may make further attempt at any site based solely on their judgment. We recorded the subsequent attempt site and also the eventual site of the first functioning intravenous catheter. We recorded the inserter’s job grade.

If the catheter was not use but still needed as a precaution after 24 hours, the catheter was flushed with 5 ml normal saline at least every 24 hours. We recorded if the catheter was used for administration of drug, fluid or blood and if additional catheter (defined as a second peripheral intravenous catheter insertion in the presence of a functioning catheter) was inserted with its indications. At removal of the last catheter (if more than one inserted), we asked the participant to rate with a VNRS (1 to 10, higher score greater satisfaction) their satisfaction with the original catheter site; a failed first attempt or subsequent catheter replacement was a priori rated the lowest score of 1. The original catheter site was inspected for the presence of swelling, bruising and vein thrombosis and palpated for tenderness. Participants were also asked on their preferred future intravenous catheterisation insertion site and to rate pain at the first insertion site with a VNRS (1 to 10, higher score more pain). These are the secondary outcomes.

Data were entered into SPSS 17 (SPSS Inc, Chicago, IL) for data analysis. We excluded those enrolled who infringed study criteria. Primary analysis was based on intention to treat. Analyses used the Student t test for comparison of means and variance, Mann Whitney U test for ordinal data or non-normally distributed data and Chi Square test for categorical datasets. Sub-analysis was also performed based on per protocol, as performed and as sited to facilitate a better understanding of the trial data and to generate hypotheses. Tests were two sided and P < 0.05 in any test was considered statistically significant.

## Results

**Figure 1:**
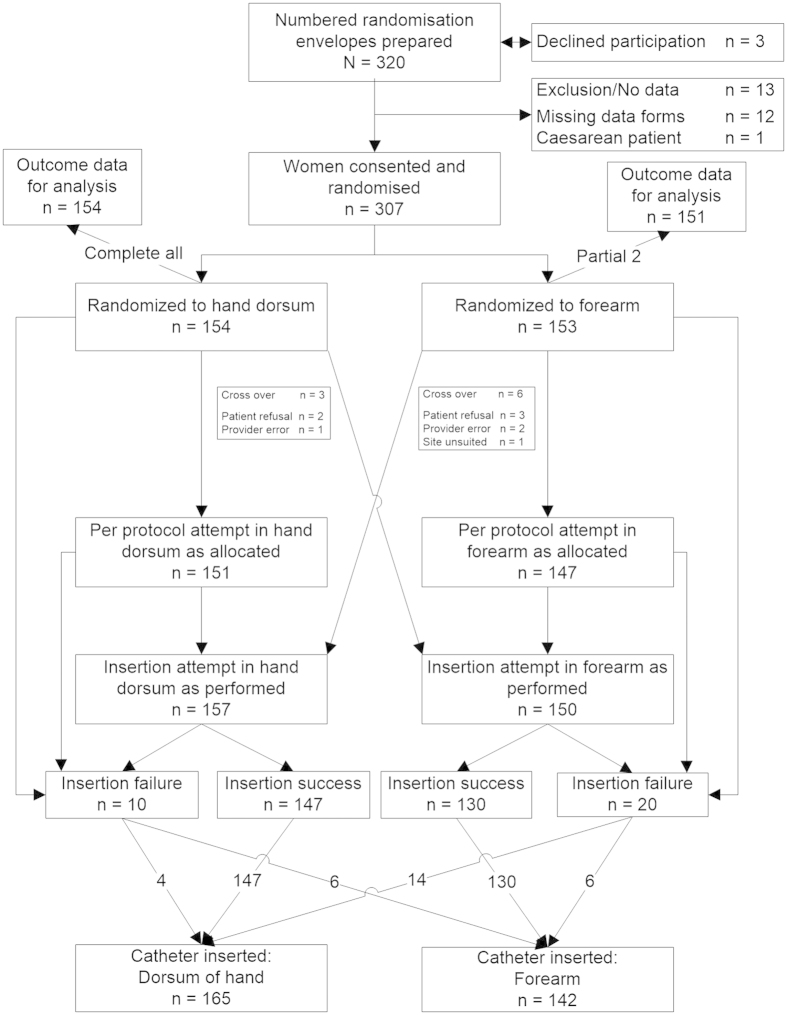
Recruitment flow chart into a randomised trial of peripheral intravenous catheterisation in obstetric patients: insertion in dorsum of the hand or lower forear.

Patients were enrolled into the trial from 18 June 2013 to 16 August 2013 and the last intravenous catheter was removed from participants on 18 August 2013. The enrolment and in-trial flow of participants is depicted in Fig. 1. We recorded three eligible women who declined participation. We stopped enrolment when all 320 numbered randomisation envelopes were used. We mistakenly enrolled one patient scheduled for Caesarean delivery– we discarded that envelope. There were 12 numbered envelopes or case report forms that could not be found; we did not replace these as we had accounted for the eventuality by preparing 320 numbered envelopes. Disregarding these 13, 154 women randomised to dorsum of the hand and 153 to forearm (total 307) were available for data analysis. Two participants randomised to forearm were not assessed after discharge from the delivery suite whilst on the postnatal ward, so their late outcome data were missing. The nine crossovers and the reasons for crossover are shown in Fig. 1. All crossovers had successful first insertions at the alternate site. All others had a per protocol first attempt at the allocated site. Following crossovers and reattempts after initial insertion failures, a first functional intravenous catheter was present in the dorsum of the hand in 165 and in the forearm in 142 participants.

The characteristics of participants (n** = **307) stratified according to intention to treat are shown in Table 1. There was no significant difference in any characteristic between the trial arms. The indications for intravenous catheter placement, participants’ site preference and the inserting inserter’s job grade were also similar.

Primary outcomes based on intention to treat analysis are shown in Table 2. Successful first insertion rates were 144/154 (93.5%) vs. 133/153 (86.7%) RR 1.1 (95% CI 1.0–1.2) P** = **0.052, insertion pain median [interquartile range] VNRS scores were 5 [3–6] vs. 4 [3–5] P** = **0.248 and replacement catheter rates were 12/154 (7.8%) vs. 11/153 (7.2%) RR 1.1 (95% CI 0.5–2.4) P** = **0.841 for dorsum of the hand and forearm respectively. As the intention to treat analysis on successful insertion showed a borderline statistical result (P** = **0.052) and the insertion success rate difference across trial arms is narrower than the pilot data, we decided to explore success rate based on per protocol as well as site specific successful insertion rate to evaluate the pure impact of insertion site; for both these sub-analyses the P values were 0.045 (NNTb 15 95% CI 7–595) and 0.040 (NNTb 15 95% CI 7–316) respectively (see Table 2) raising the possibility of Type 2 statistical error with the intention to treat analysis. There was no difference in insertion pain score or the need for catheter replacement in the sub-analyses. Sub-analyses based on first successful catheter dwell site which offered the clearest observational data of dwelling site impact on catheter malfunction was also done; this showed no difference across trial arms.

Table 3 shows the secondary outcome measures across trial arms. There was no difference across trial arms in the reasons underlying catheter malfunctions necessitating replacements, provider perception on technical difficulty of insertion, patient satisfaction regarding catheter site (VNRS score), catheter usages, need and indications for additional catheters, future catheterisation site fidelity rates, presence of swelling, bruising, tenderness, vein thrombosis or pain (VNRS scores) at original catheter site and time interval between insertion and last catheter removal.

Post hoc, we calculated that our trial’s statistical power is only 49.4% (calculated using https://www.dssresearch.com/KnowledgeCenter/toolkitcalculators/statisticalpowercalculators.aspx) given the smaller insertion success gap in our trial of 93.5% vs. 86.9% compared with pilot data success rates of 89% vs. 74% for dorsum of the hand and forearm respectively. The job grade (denoting clinical experience) of the inserting provider also did not show any significant difference on post hoc analysis; house officers had insertion success of 92.2% and 87.2% (P** = **0.224) and the more experienced medical officers had success rates of 100% and 85% (P** = **0.080) respectively for hand dorsum and forearm vein attempts respectively. Medical officers had a very similar failure rate to house officers at forearm attempts, plausibly due to the lower forearm being a technically more challenging site which was not surmounted by greater experience. We then divided the trial period into an early phase and a late phase (i.e. catheter insertion on or after 5 July 2013; with approximately equal distribution of participant number to each phase) to investigate whether providers were negotiating the learning curve. Dorsum of the hand insertion success rates were 77/80 (96.3%) vs. 70/77 (90.9%) RR 1.1 95% CI 1.0–1.2 P** = **0.171 and forearm success rates were 61/71 (85.9%) vs. 69/79 (87.3%) RR 1.0 95% CI 0.9–1.1 P** = **0.798 for early vs. late trial phases respectively. There is no suggestion of a significant learning curve. We were not aware of significant unintended harm from the interventions.

## Discussion

Our finding indicate that the success rate for inserting an intravenous catheter in a dorsum of the hand vein compared to lower forearm vein might be higher; all other findings including insertion pain, catheter malfunction and replacement, additional catheter, patient satisfaction, post-removal catheter site complications or pain and future fidelity to the allocated site were similar. Our pilot data indicates that in routine practice, junior providers on our delivery suite would preferentially insert the intravenous catheter by a ratio of 81 to 19 into the dorsum of the hand compared to forearm. On the converse, at enrolment 63.8% of participants in our trial had no preference on insertion site but where a preference was stated 25.9% preferred the dorsum of the hand (vs.10.4% for forearm). The totality of our findings would indicate that less experienced providers on the delivery suite should be allowed to insert a catheter at the dorsum of the hand as that site was also slightly preferred by women.

We performed a PubMed (http://www.ncbi.nlm.nih.gov/pubmed) search on 2 April 2015 using terms [(intravenous) AND (catheter OR cannula OR catheterization OR catheterization OR cannulation)] AND (hand and forearm) without any restrictions which retrieved 55 items. None of the items returned was a prospective comparative trial of catheter insertion at dorsum of the hand versus at the forearm. There is a paucity of direct trial data on the relative merits of these two commonest sites for peripheral intravenous catheterisations though secondary analysis of a trial’s data has indicated that cannulation success was not associated with insertion site^19^.

The report of secondary data of a trial of 3,283 adult medical and surgical patients (5,907 catheters) with a peripheral intravenous catheter with greater than 4 days of expected use shows catheter occlusion and accidental removal risks are with higher with hand compared with forearm sites hazard ratio HR 1.47 95% CI 1.28–1.68 and HR 2.45 95% CI 1.92–3.10 respectively. The report concludes that peripheral intravenous catheter survival is improved by forearm insertion^22^. In our trial with median catheter dwell time of only one day in young and healthy women admitted through the delivery suite, there was no difference in occlusion rate and no recorded case of catheter replacement caused by accidental removal.

The forearm catheter site has been found to be an independent predictor for peripheral vein thrombosis (HR 1.93 95% CI 1.20–3.01)^23^. Another study finds that the highest incidence of peripheral intravenous cannulae thrombophlebitis was found in patients with cannulae inserted in the dorsal side of the hand veins compared to those with cannulae inserted in cubital fossa veins (OR:3.33; CI:1.37–8.07; P < 0.001)^23^. For severe phlebitis, hand to forearm relative risk is 0.71 and wrist to forearm relative risk is 0.60^24^ indicating the lowest crude risk at the wrist. These findings on catheter site related thrombophlebitis are mixed and heterogeneous. Our trial showed similar rates for catheter site swelling, tenderness, pain or vein thrombosis in the dorsum of the hand or the lower forearm.

In a study of 500 peripheral intravenous catheters insertions, the most marked effect on longevity was with 18G placed in the forearm/wrist (median 72 hours) whilst a 22G intravenous catheter placed in the hand had a median lifespan of 29 hour. The study concludes that 18G catheters placed in the forearm/wrist should be attempted in patients who require sustained cannulation^25^. In our trial, catheter replacement due to malfunction of the 18G catheter was minimally different across the trial arms (7.8% vs. 7.2% for dorsum of the hand vs. lower forearm respectively).

In a single center study of complications of peripheral intravenous catheters in the hand and forearm over a 3-year period more than 50% of major complications occurred in the hand with the authors concluding that the hand is a common site for minor and major intravenous catheter complications^26^. In another study of 254 patients undergoing elective surgery, the dorsum of the hand was the site with the least number of complications and gauge of cannula had no influence on the rate of complications^27^. These data on catheter site complications is evidently inconsistent probably due to confounding arising from heterogeneity in the different study populations and their clinical circumstances. In our trial of obstetric patients on the delivery suite, there was no suggestion of an appreciable difference in catheter site related complications.

Our trial has strengths and limitations. We had few women who declined to participate, low number of crossovers, very high per protocol intervention and little missing outcome data. There did not appear to be any confounding from a within the trial learning curve as success rates at both sites were similar in the first and second halves of the trial. We performed a powered (beta 0.1) study based on local and contemporary pilot data. However, due to the smaller than anticipated difference in the catheter insertion success rates across intervention arms within the trial compared to during pilot data collection, statistical power was reduced to only 49.4% on this outcome. Our interventions were unmasked but given the generally higher insertion success rates within the trial compared to pilot data for both trial interventions, there was no indication of provider bias in making their best effort. We believe our findings are internally valid and generalisable to other delivery suite practices with similar provision for peripheral intravenous catheterisation.

## Conclusion

In obstetrics patients on the delivery suite who needed a peripheral intravenous catheter, both the dorsum of the hand and lower forearm are viable insertion sites; if a dorsum of the hand vein is available and less experienced providers prefer to insert there, they may do so based on our finding.

## Additional Information

**How to cite this article**: Tan, P. C. *et al*. Peripheral Intravenous Catheterisation in Obstetric Patients in the Hand or Forearm Vein: A Randomised Trial. *Sci. Rep*. **6**, 23223; doi: 10.1038/srep23223 (2016).

## Figures and Tables

**Table 1:** Characteristics of trial participants stratified according to treatment allocation (intravenous catheterisation on dorsum of hand or lower forearm).

	Hand Dorsum n = 154	Forearm n = 153
Age (years)	29.8 ± 3.8	29.7 ± 3.8
Gestational age (weeks)	39.3 ± 1.1	39.3 ± 1.2
Body Mass Index	28.4 ± 4.8	28.7 ± 4.7
Parity		
Nulliparous	70 (45.5)	66 (43.1)
Para 1	39 (25.3)	47 (30.7)
Para 2 and above	45 (29.2)	40 (26.1)
Ethnicity		
Malay	110 (71.4)	95 (62.1)
Chinese	22 (14.3)	30 (19.6)
Indian	14 (9.1)	12 (7.8)
Others	8 (5.2)	16 (10.5)
Handedness^2^		
Right	138 (89.6)	139 (90.8)
Left	16 (10.4)	14 (9.2)
Anticipated use of intravenous catheter^1^		
Blood taking	144 (93.5)	143 (93.5)
Intravenous fluid	65 (42.2)	74 (48.4)
Precautionary	49 (31.8)	46 (30.3)
Intravenous drug	23 (14.9)	23 (15.0)
Blood transfusion	5 (3.2)	2 (1.3)
Participants’ preferred site (at enrolment)^2^		
Dorsum of hand	45 (29.2)	34 (22.2)
Forearm	14 (9.1)	18 (11.8)
No preference	95 (61.7)	101 (66.0)
Non-dominant arm first attempt	118 (76.6)	124 (81.0)
Inserter experience^3^		
House officer	129 (83.8)	133 (86.9)
Medical officer	25 (16.2)	20 (13.1)
Participants undelivered at hospital discharge	5 (3.2)	10 (6.6)

Data expressed as mean ± standard deviation and number (%). Analyses by Student t test for comparison of means for continuous data, Fisher’s exact test for 2 × 2 categorical datasets and Chi Square test for larger than 2 × 2 categorical datasets. P > 0.05 for all analyses.

^1^Providers may provide more than one anticipated use for intravenous cannulation.

^2^As stated by participants.

^3^Preregistration house officers participating in the trial have a minimum 6 months post basic qualification experience and were independently inserting peripheral intravenous cannulas. Medical officers were registered medical practitioners and all were in the obstetrics and gynaecology specialist trainee program.

**Table 2:** Main outcomes after randomisation to dorsum of hand or forearm intravenous catheterization.

	Hand Dorsum n = 154	Forearm n = 153	Relative Risk (95% Confidence Interval)	P value	NNTb (95% CI)^4^
Successful first catheterisation^1^ (intention to treat)	144 (93.5)	133 (86.9)	1.1 (1.0–1.2)	P** = **0.052	
Successful first catheterisation^1^ (per protocol attempt excluding crossovers)	141 (93.4) n** = **151	127 (86.4) n** = **147	1.1 (1.0–1.2)	P** = **0.045	15 (7–597)
Successful first catheterisation^1^ (per insertion site attempt including crossovers)	147 (93.6) n** = **157	130 (86.7) n** = **150	1.1 (1.0–1.2)	P** = **0.040	15 (7–316)
Insertion pain VNRS score^2^ (intention to treat)	5 [3–6] 4.5 ± 2.1	4 [3–5] 4.2 ± 2.1		P** = **0.248 P** = **0.239	
Insertion pain VNRS score^2^ (per protocol attempt excluding crossovers)	5 [3–6] 4.4 ± 2.1 n** = **151	4 [2–5] 4.1 ± 2.1 n** = **147		P** = **0.239 P** = **0.250	
Insertion pain VNRS score^2^ (per insertion site attempt including crossovers)	5 [3–6] 4.4 ± 2.1 n =157	4 [2–5] 4.2 ± 2.1 n** = **150		P = 0.252 P** = **0.282	
Catheter replacement/malfunction^3^ (intention to treat)	12 (7.8)	11 (7.2)	1.1 (0.5–2.4)	P** = **0.841	
Catheter replacement/malfunction^3^ (per protocol successes only; crossovers and first attempt failures excluded)	11 (7.8) n** = **141	8 (6.2) n** = **129	1.3 (0.5–3.0)	P** = **0.608	
Catheter malfunction/replacement^3^ (as sited successfully)	12 (7.3) n** = **165	11 (7.7) n** = **142	0.9 (0.4–2.1)	P** = **0.875	

Data expressed as number (%), mean ± standard deviation and median [interquartile range]. Analysis is by Fisher’s exact test for categorical datasets, Mann Whitney U test for ordinal data and Student t test for comparison of means and variance.

^1^Defined as full placement of the catheter into a vein after a single skin break confirmed with a 5 ml saline flush.

^2^Pain VNRS (visual numerical rating score) 1 to 10 (lower score, less pain) at first catheterisation attempt.

^3^Defined as the need for further catheterisation prior to hospital discharge due to unsatisfactory performance of the first indwelling catheter.

^4^Number needed to treat to benefit and 95% confidence interval.

**Table 3:** Secondary outcomes after randomisation to dorsum of hand or forearm intravenous catheterisation (intention to treat).

	Hand Dorsumn = 154	Forearm n = 153	Relative Risk (95% Confidence Interval)	P value
*Indications for catheter replacement*^1^				
Blocked/too slow	6	8		
Site painful	7	6		
Site swollen	4	8		
Catheter displaced	1	0		
	n** = **12^1^	n** = **11^1^		
*Insertion success* (*inserter experience*)^2^				
House officer (n** = **252)	119/129 (92.2)	116/133 (87.2)	1.1 (1.0–1.1)	P** = **0.224
Medical officer (n** = **45)	25/25 (100)	17/20 (85.0)	1.2 (1.0–1.4)	P** = **0.080
*Insertion difficulty*				P** = **.117
Straightforward	140 (90.9)	131 (85.6)		
Moderate	4 (2.8)	2 (1.3)		
Failed	10 (6.5)	20 (13.1)		
VNRS satisfaction score with catheter site^3^	7 [5–9]	7 [5–8]		P** = **0.914
*Catheter used for*				
Blood taking	146 (94.8)	143 (93.5)	1.3 (0.5–3.3)	P** = **0.637
Intravenous drug	54 (35.1)	50 (32.7)	1.1 (0.7–1.8)	P** = **0.718
Intravenous infusion	129 (83.8)	116 (75.8)	1.6 (0.9–2.9)	P** = **0.090
Additional catheter^4^	6 (3.9)	6 (3.9)	1.0 (1.0–1.0)	P =0.991
*Indication(s) for additional catheter*^1^				
Anaesthetist in operating theatre	5	4		
Patient unstable	1	2		
Additional fluid infusion	1	1		
Unascertained	1	0		
	n** = **6	n** = **6		
*Patient preference for future catheter site* (after catheter removal)				P = 0.470
Same site as allocated	78 (50.6)	66 (43.7)		
Other site to allocated	36 (23.1)	39 (25.8)		
No preference	40 (26.3)	46 (30.5)		
*Insertion site complications*^5^				
Swelling	13 (8.4)	8 (5.3)	1.6 (0.7–4.1)	P** = **0.367
Bruising	5 (3.2)	3 (2.0)	1.7 (0.4–7.1)	P** = **0.723
Tenderness	17 (11.0)	14 (9.3)	1.2 (0.6–2.6)	P** = **0.706
Thrombosed vein	4 (2.6)	1 (0.7)	4.0 (0.4–35.3)	P** = **0.371
Pain VNRS	2 [1–3]	2 [1–3]		P** = **0.955
Insertion to removal interval (days)	1 [1,2]	1 [1, 2]		P** = **0.443

Data expressed as number (%) and median [interquartile range]. Analysis is by Fisher’s exact test for categorical datasets, and Mann Whitney U test for ordinal data.

^1^Providers may state multiple malfunctions for each catheter replacement or for each additional catheter placement.

^2^Preregistration house officers participating in the trial have a minimum 6 months post basic qualification experience and were independently inserting peripheral intravenous cannulas. Medical officers were registered medical practitioners and all were in the obstetrics and gynaecology specialist trainee program.

^3^Visual numerical rating score (VNRS – scored 1 to 10, high score greater satisfaction) taken after final catheter removal. A prior decision to give satisfaction score of 1 (lowest) if first insertion failed or a replacement catheter was needed.

^4^Insertion of a second catheter in the presence of a functioning indwelling catheter.

^5^At site of first catheterization.

## References

[b1] Webster J., Osborne S., Rickard C. M. & New K. Clinically-indicated replacement versus routine replacement of peripheral venous catheters. The Cochrane database of systematic reviews 4, CD007798, doi: 10.1002/14651858.CD007798.pub3 (2013). 20238356

[b2] Kumar M., Vandermeer B., Bassler D. & Mansoor N. Low-dose heparin use and the patency of peripheral IV catheters in children: a systematic review. Pediatrics 131, e864–872, doi: 10.1542/peds.2012-2403 (2013). 23439893

[b3] Flint A., McIntosh D. & Davies M. W. Continuous infusion versus intermittent flushing to prevent loss of function of peripheral intravenous catheters used for drug administration in newborn infants. The Cochrane database of systematic reviews, CD004593, doi: 10.1002/14651858.CD004593.pub2 (2005). PMC12586969 16235368

[b4] Shah P. S., Ng E. & Sinha A. K. Heparin for prolonging peripheral intravenous catheter use in neonates. The Cochrane database of systematic reviews, CD002774, doi: 10.1002/14651858.CD002774.pub2 (2005). 16235300 PMC13120738

[b5] Niel-Weise B. S., Stijnen T. & van den Broek P. J. Should in-line filters be used in peripheral intravenous catheters to prevent infusion-related phlebitis? A systematic review of randomized controlled trials. Anesthesia and analgesia 110, 1624–1629, doi: 10.1213/ANE.0b013e3181da8342 (2010). 20435946

[b6] Gillies D. . Optimal timing for intravenous administration set replacement. The Cochrane database of systematic reviews, CD003588, doi: 10.1002/14651858.CD003588.pub2 (2005). 16235329

[b7] Lysakowski C., Dumont L., Tramer M. R. & Tassonyi E. A needle-free jet-injection system with lidocaine for peripheral intravenous cannula insertion: a randomized controlled trial with cost-effectiveness analysis. Anesthesia and analgesia 96, 215–219, table of contents (2003). 12505955 10.1097/00000539-200301000-00044

[b8] Uman L. S. . Psychological interventions for needle-related procedural pain and distress in children and adolescents. The Cochrane database of systematic reviews 10, CD005179, doi: 10.1002/14651858.CD005179.pub3 (2013). 24108531

[b9] Masud S. . Contribution of a heating element to topical anesthesia patch efficacy prior to vascular access: results from two randomized, double-blind studies. Journal of pain and symptom management 40, 510–519, doi: 10.1016/j.jpainsymman.2010.01.022 (2010). 20678893

[b10] Hijazi R., Taylor D. & Richardson J. Effect of topical alkane vapocoolant spray on pain with intravenous cannulation in patients in emergency departments: randomised double blind placebo controlled trial. Bmj 338, b215, doi: 10.1136/bmj.b215 (2009). 19208703 PMC2640112

[b11] Prien T. Intradermal anaesthesia: comparison of several compounds. Acta anaesthesiologica Scandinavica 38, 805–807 (1994). 7887101 10.1111/j.1399-6576.1994.tb04008.x

[b12] Vaghadia H., al-Ahdal O. A. & Nevin K. EMLA patch for intravenous cannulation in adult surgical outpatients. Canadian journal of anaesthesia = Journal canadien d’anesthesie 44, 798–802 (1997). 10.1007/BF03013153 9260005

[b13] Cooper J. A., Bromley L. M., Baranowski A. P. & Barker S. G. Evaluation of a needle-free injection system for local anaesthesia prior to venous cannulation. Anaesthesia 55, 247–250 (2000). 10671842 10.1046/j.1365-2044.2000.01210.x

[b14] Suren M. . Comparison of the use of the Valsalva maneuver and the eutectic mixture of local anesthetics (EMLA(R)) to relieve venipuncture pain: a randomized controlled trial. Journal of anesthesia 27, 407–411, doi: 10.1007/s00540-012-1540-1 (2013). 23242570

[b15] Webster J., Gillies D., O’Riordan E., Sherriff K. L. & Rickard C. M. Gauze and tape and transparent polyurethane dressings for central venous catheters. The Cochrane database of systematic reviews, CD003827, doi: 10.1002/14651858.CD003827.pub2 (2011). 22071809

[b16] Lenhardt R., Seybold T., Kimberger O., Stoiser B. & Sessler D. I. Local warming and insertion of peripheral venous cannulas: single blinded prospective randomised controlled trial and single blinded randomised crossover trial. Bmj 325, 409–410 (2002). 12193353 10.1136/bmj.325.7361.409 PMC119431

[b17] Meyer B. A., Little C. J., Thorp J. A., Cohen G. R. & Yeast J. D. Heparin versus normal saline as a peripheral line flush in maintenance of intermittent intravenous lines in obstetric patients. Obstetrics and gynecology 85, 433–436, doi: 10.1016/0029-7844(94)00409-7 (1995). 7862386

[b18] Niesen K. M., Harris D. Y., Parkin L. S. & Henn L. T. The effects of heparin versus normal saline for maintenance of peripheral intravenous locks in pregnant women. Journal of obstetric, gynecologic, and neonatal nursing: JOGNN/NAACOG 32, 503–508 (2003). 10.1177/0884217503255203 12903700

[b19] Robinson P. A., Carr S., Pearson S. & Frampton C. Lignocaine is a better analgesic than either ethyl chloride or nitrous oxide for peripheral intravenous cannulation. Emergency medicine Australasia: EMA 19, 427–432, doi: 10.1111/j.1742-6723.2007.01008.x (2007). 17919215

[b20] Dupont W. D. & Plummer W. D. Jr. Power and sample size calculations. A review and computer program. Controlled clinical trials 11, 116–128 (1990). 2161310 10.1016/0197-2456(90)90005-m

[b21] Hendry F., Checketts M. R. & McLeod G. A. Effect of intradermal anaesthesia on success rate and pain of intravenous cannulation: a randomized non-blind crossover study. Scottish medical journal 56, 210–213, doi: 10.1258/smj.2011.011160 (2011). 22089042

[b22] Wallis M. C. . Risk factors for peripheral intravenous catheter failure: a multivariate analysis of data from a randomized controlled trial. Infection control and hospital epidemiology 35, 63–68, doi: 10.1086/674398 (2014). 24334800

[b23] Mestre Roca G. . Assessing the influence of risk factors on rates and dynamics of peripheral vein phlebitis: an observational cohort study. Medicina clinica 139, 185–191, doi: 10.1016/j.medcli.2011.12.021 (2012). 22538061

[b24] Maki D. G. & Ringer M. Risk factors for infusion-related phlebitis with small peripheral venous catheters. A randomized controlled trial. Annals of internal medicine 114, 845–854 (1991). 2014945 10.7326/0003-4819-114-10-845

[b25] Dillon M. F. . Factors that affect longevity of intravenous cannulas: a prospective study. QJM: monthly journal of the Association of Physicians 101, 731–735, doi: 10.1093/qjmed/hcn078 (2008). 18621805

[b26] Kagel E. M. & Rayan G. M. Intravenous catheter complications in the hand and forearm. The Journal of trauma 56, 123–127, doi: 10.1097/01.TA.0000058126.72962.74 (2004). 14749578

[b27] Holland R. B., Levitt M. W., Steffen C. M. & Lipski P. S. Intravenous cannulas. Survey of their use in patients undergoing elective surgery. The Medical journal of Australia 2, 86–89 (1982). 7121369

